# Minor symptoms: the illnesses of the 21^st^ Century

**DOI:** 10.1590/S1516-31802001000200001

**Published:** 2001-03-02

The 20^th^ Century was devoted to combating the world's most lethal diseases, including cardiovascular disease and cancer. The reason for this is obvious. These are the most important causes of mortality in the world as a whole.^1^ However, at the end of the century, some attention was also being directed towards minor symptoms. What are these?

The first time I saw this expression, it caught my attention. In fact, it was coined by Anthony Komaroff, a researcher from Brigham and Women's Hospital at Harvard Medical School, to describe a range of symptoms like headache, low back pain, fatigue, cough and others that are not exactly diseases.^2^ Most of the time it is difficult to find any alteration upon physical examination. They are basically symptoms, but they are the most important causes of morbidity in outpatient services.

Most of the demands placed on medical services are caused by minor symptoms. However, the majority of guidelines and evidence-based medicine are preoccupied with major symptoms and their consequences.

When a person has an acute thoracic pain, one strong possibility is a heart attack. Physicians are very concerned about such patients and give them all their attention, as this condition frequently occurs with possibly lethal diseases. In an emergency service, the patient with acute thoracic pain has total priority. This is easy to understand: anyone over a certain age could get into such a situation, and its lifethreatening nature causes compassion, so it is important to give this person the best attention.

In contrast, we can imagine a person with headache: a woman who has had migraine headaches since she was 18. Now she is 42, with pain almost every day. She uses a lot of medications, yet continues to have pain all the time. Physicians are not very impressed with these cases. They are not life-threatening, but people with these symptoms have a lot of difficulty in coping with them. Frequently, such patients create misunderstanding in the emergency room because they want more attention, while all the attention is being directed towards lethal diseases.

Whereas guidelines for treatment of acute myocardial infarction and other emergencies have been available for many years, the guidelines for minor symptoms are only now being drawn up and some strategies still need to be verified using appropriate clinical trials. However, there is another important point at work here: little money is available for research into minor symptoms.

In my view, the greatest problem in dealing with patients complaining of minor symptoms is that physicians are not well trained to treat these symptoms, which are not an important part of medical education. Another point is that minor symptoms are frequently associated with psychiatric co-morbidity and again, most physicians do not have the grounding to make psychiatric diagnoses. This occurs most frequently when psychiatric patients focus their complaints on somatic manifestations. It is known that fifty per cent of patients seeking medical consultation with fatigue as the main complaint have depression as the final diagnosis. And the same is common among other chronic pain syndromes.

Another important point is the cost of minor symptoms. Frequently, this type of patient goes to physicians many times, looking for a diagnosis that is not made. Thus, these patients circulate among several physicians, and are submitted to a lot of unnecessary diagnostic tests before the correct diagnosis or the correct treatment is recommended.

There is a lot of data from around the world about the burden of minor symptoms. But we also have a lot of data in Brazil. Bigal et al showed that migraine was an important public health problem in the clinical hospital.^3^ Vincent et al showed that headache pain interfered with work productivity in 10% of the subjects, corresponding to 538.75 hours off work, with an estimate annual cost for each headache of US$ 125.98 per employee. ^4^

At the beginning of this new century, questions about the quality of life have come up for discussion. For countries like Brazil and others from Latin America, this is the moment to pay attention not only to questions of quantity of life, i.e. it is not sufficient just to be alive. It is also necessary to be alive with a good quality of life. Everyone has to live well and not just live.

What is needed to achieve this? First, it is necessary to disseminate how to deal with minor symptoms and how to diagnosis psychiatric problems. Family physicians and emergency doctors have to be prepared to cope with these situations. Patients with chronic minor symptoms are frequently problematic patients, who suffer a lot of discomfort and have been circulating through the medical system without treatment for a long time. Second, it is time to forget prejudices about quality of life. Quality of life is not a luxury, but an essential condition that has to be present in the lives of all people in the world.
